# Study protocol for Enhancing Parenting In Cancer (EPIC): development and evaluation of a brief psycho-educational intervention to support parents with cancer who have young children

**DOI:** 10.1186/s40814-017-0215-y

**Published:** 2017-12-11

**Authors:** Lesley Stafford, Michelle Sinclair, Jane Turner, Louise Newman, Claire Wakefield, Mei Krishnasamy, G. Bruce Mann, Leslie Gilham, Kylie Mason, Paula Rauch, Julia Cannell, Penelope Schofield

**Affiliations:** 1 Centre for Women’s Mental Health, Royal Women’s Hospital, Parkville, Victoria Australia; 2 School of Psychological Sciences, University of Melbourne, Melbourne, Victoria Australia; 3 Discipline of Psychiatry, University of Queensland, Herston, Queensland Australia; 4 Royal Brisbane and Women’s Hospital, Brisbane, Queensland Australia; 5 Department of Psychiatry, University of Melbourne, Melbourne, Victoria Australia; 6 School of Women’s and Children’s Health, University of New South Wales, Sydney, New South Wales Australia; 7 Behavioural Sciences Unit, Kids Cancer Centre, Sydney Children’s Hospital, Randwick, New South Wales Australia; 8 Department of Nursing, University of Melbourne, Melbourne, Victoria Australia; 9 Department of Cancer Experiences Research, Peter MacCallum Cancer Centre, Parkville, Victoria Australia; 10 Breast Service, Victorian Comprehensive Cancer Centre, Parkville, Victoria Australia; 11 Department of Surgery, University of Melbourne, Melbourne, Victoria Australia; 12 Parkville Integrated Haematology Service, Victorian Comprehensive Cancer Centre, Parkville, Victoria Australia; 13 Department of Medicine, University of Melbourne, Melbourne, Victoria Australia; 14 Department of Psychiatry, Massachusetts General Hospital, Boston, MA USA; 15 Department of Psychology, Swinburne University, Hawthorn, Victoria Australia

**Keywords:** Cancer, Oncology, Parenting, Intervention, Psycho-education

## Abstract

**Background:**

Parents with cancer have high rates of psychological morbidity, and their children are at risk of poor psychosocial outcomes, particularly in the context of parental distress and poor family communication. Parents express concerns about the impact of cancer on their children and report a lack of professional guidance in meeting their children’s needs. Few parenting interventions exist and current interventions have extensive infrastructure demands making them unsuitable for routine use in most health settings. The aims of this study are to develop and establish the feasibility and acceptability of a novel and accessible psycho-educational intervention to improve parenting efficacy and decrease parental stress among adults with cancer who have children aged 3–12 years. The intervention will be suitable for parents with cancer who are receiving treatment with a view to longer term survival, irrespective of cancer diagnosis, and their respective co-parents.

**Methods/design:**

This study comprises two phases using the UK Medical Research Council framework for developing complex interventions. In the development phase, intervention content will be iteratively developed and evaluated in consultation with consumers, and in the piloting phase, feasibility will be tested in a clinical sample of 20 parents with cancer and their co-parents using a single arm, pre-test post-test design. The intervention will comprise an audiovisual resource (DVD), a question prompt list, and a telephone call with a clinical psychologist. Questionnaires administered pre- and 1 month post-intervention will assess parental stress, psychological morbidity, quality of life, self-efficacy and perceptions of child adjustment, and family functioning. Intervention feasibility will be determined by mixed-method participant evaluation of perceived usefulness, benefits, and acceptability.

**Discussion:**

This new initiative will translate existing descriptive evidence into an accessible intervention that supports parenting during cancer treatment and meets the information needs of parents with cancer and their families. This is an important advance: despite increasing recognition of the impact of parental cancer on the family, intervention research lags behind the descriptive literature. This low-intensity, accessible, and targeted intervention places minimal burden on infrastructure and promotes patient autonomy and self-management. If feasible, this style of intervention may be a template for future interventions with similar populations.

## Background

For adults with young children, parenting involves the daily provision of physical and emotional care and support. Parenting young children is a primary and essential activity in which the attachment figure and the quality of the attachment relationship have a significant role in child developmental outcomes [1]. Parental cancer poses unique challenges to these families as parents must balance the demands of managing their illness with fulfillment of their caregiving responsibilities. Many parents do not know how best to meet their children’s emotional needs or communicate with them about the cancer diagnosis and treatment. Parents have reported feeling overwhelmed by the effort of maintaining routine at home as a way of protecting their children and may feel guilty about not being a “good” parent [2]. Parents have also reported that they are not well supported by oncology professionals in this regard and that they would benefit from clearer guidance and advice about parenting during cancer [2, 3]. For parents with cancer, parenting concerns may constitute a substantial source of stress [4, 5]. This occurs in the context of the already considerable disease burden associated with cancer including living with uncertainty about one’s future, undergoing possibly painful and protracted treatments, and high rates of psychological morbidity [6, 7]. In families with two parents, co-parents may also experience substantial stress, reduced quality of life [8, 9], and decline in parenting efficacy [5]. In addition to providing emotional care and physical support for the parent with cancer, co-parents are often required to assume the ill parents’ role as well as their own. Co-parents may also struggle with meeting the often competing needs of their children and ill partner.

Children with a parent with cancer are at increased risk of poor psychosocial outcomes [10–12], particularly internalizing problems such as anxiety and depression [11]. This is consistent with the threat of loss inherent in a cancer diagnosis. Parental cancer threatens the availability of the attachment figure for the child which may be compounded by separation due to hospitalization, decreased parental availability, and disruption of usual roles and routines. Children may also become parentified, i.e., they may prematurely take on more adult roles [13]. Often, parents are unaware of their children’s problems in relation to the cancer [14]. Indeed, children report that their information and emotional needs are poorly met [13]. This is significant because children’s anxiety levels appear to be associated with whether and how they are informed of their parent’s cancer, with well-informed children demonstrating better adjustment [15]. Psychological distress and impaired functioning depend on many factors including the child’s age and gender, the gender of the ill parent, parental attachment, family dynamics and cohesion, and whether the family unit is intact [11, 16]. Family and parenting variables, particularly, communication, the quality of the parental relationship, and parental psychological morbidity are the most consistent predictors of child psychosocial outcomes [11, 16, 17].

Parents with cancer, especially those with greater illness- and treatment-related effects [17] and poorer quality of life, believe that their children are adversely affected by cancer-related parenting changes [5]. These observations are consistent with findings that parents’ psychological and physical functioning do impact on children’s emotional functioning [17–19]. Further, parental beliefs about their capacity or efficacy to parent effectively are also associated with child adjustment [20, 21]. One pathway linking parental illness to child distress is through the disruption in parenting brought about by changes in routine, reduced physical and emotional availability, and poor communication [18, 21–23].

Despite increasing recognition of the impact of parental cancer on the family unit, intervention research lags behind the descriptive literature [24]. A small number of interventions aimed at children, families, or parents in the context of parental cancer have been developed and evaluated [25]. Only four distinct parenting interventions in which the parent with cancer is the target of the intervention have been published [21, 26–28]. In a randomized trial, mothers with breast cancer who had a child between the ages of 8 and 12 years received five, 1-h individual face-to-face educational sessions at two weekly intervals [21]. In a second study, 24 families in which one parent had cancer participated in a needs-based counseling intervention of 5–6 sessions delivered in the home setting over a period of 3–10 months [26]. In another intervention, mothers with breast cancer and their children received a 3-week multidisciplinary inpatient intervention [27]. Finally, 20 parents participated in 2-day-long psycho-educational workshops focused on identifying children’s needs and communicating with children about cancer [28]. With the exception of the aforementioned workshop-based intervention which was not formally evaluated, all interventions yielded improvements in aspects of parental psychological morbidity, family functioning, parenting skills, and child adjustment, but with their extensive infrastructure demands, none of these interventions are appropriate for routine use.

Consequently, we propose to develop and evaluate a novel and accessible, psycho-educational intervention to improve parenting efficacy and promote family communication, thereby decreasing parental stress and psychological morbidity as well as enhancing children’s psychosocial adjustment. The content of the intervention will be delivered via three components comprising a psycho-educational DVD, a question prompt list (QPL), and a telephone call supplemented by additional referrals/written resources, if needed.

Derived from attachment [1] and social cognitive theory [29], as well as clinical experience, this new intervention will address parental anxiety about maintaining their role as attachment figure and provide strategies to improve family communication and maintain empathic relationships. The intervention will translate existing descriptive evidence into an accessible intervention that supports parenting during cancer treatment and meets the information and emotional needs of parents and families.

### Aims

The aims of this study are twofold: The first aim is to develop a novel psycho-educational intervention to support individuals with cancer who are parents to children aged 3–12 years. The second aim is to determine the feasibility and acceptability of our intervention. The intervention will be appropriate for parents diagnosed with any cancer, where treatment is delivered with curative intent or with a view to longer term survival, and their respective co-parents. Evidence of potential to benefit parental stress and self-efficacy, psychological morbidity, quality of life, family functioning, and perceptions of child adjustment will be explored as a component of the pilot phase.

## Methods/design

This intervention will be developed in accordance with the UK Medical Research Council (MRC) Framework [30] for developing complex interventions. This framework describes a flexible, iterative development and testing process and was selected because it addresses common difficulties in program complexity. The framework comprises four elements, but this project comprises only the first two phases:

1. A development phase in which the content of the intervention will be iteratively developed and evaluated in consultation with consumers on the study steering committee
2. A piloting phase in which the intervention will be tested to establish feasibility and acceptability in a clinical sample of 20 parents with cancer and their co-parents using a single arm, pre-test post-test design.

Phase 1 of the MRC framework comprises *developing the intervention* which will comprise a psycho-educational DVD, followed by a QPL, followed by a telephone call with the provision of additional resources/referrals, if appropriate. A QPL is a structured list of questions that a patient may wish to ask his or her health professional about their illness or treatment. Evidence suggests that QPLs are useful tools for cancer patients, empowering them to ask specific questions that might not otherwise have been asked [31]. Intervention content and delivery are described in more detail below.

Phase 1 involves the following steps:

- A review of the literature relating to parenting with cancer. This literature review, which has been completed, included, within the parameters of the population of interest, the psychosocial impact of parental cancer on parents, families, and children; determinants of psychosocial outcomes among parents, children, and families; and interventions that target parents directly (rather than children or families).
- The findings of the literature review will be used to guide the development of a semi-structured interview schedule to be administered by telephone to two groups: (i) oncology health professionals working with parents with cancer and (ii) parents with a previous diagnosis of cancer and their co-parents, if appropriate. These interviews will be used to verify the relevance of the empirical literature reviewed in (a) to the local population and assess consumers and health professionals’ needs and preferences regarding the content of a new intervention to support parents with cancer. The oncology health professionals (target *n* = 10) will be recruited from participating hospitals. The consumers to be interviewed (target *n* = 15) will be recruited with the assistance of several community-based oncology advocacy groups who are collaborating on this study.
- Telephone interviews will be audiorecorded and transcribed verbatim. Transcripts will be analyzed using the Framework method [32], a well-established, systematic method of organizing and categorizing qualitative data.
- Guided by attachment [1] and social cognitive theory [29], as well as the literature review and the data from the telephone interviews, the specific content (script) of the DVD will be iteratively developed in a collaborative process involving the multidisciplinary research team, consumers on the study steering committee, and a professional production company. The ongoing consultation and engagement with consumers will enhance the relevance and acceptability of the intervention material and ensure that content meets consumer needs. DVD development will be based on a best practice framework for creating audiovisual material in these settings [33]. In this framework, emphasis is placed on maximizing audience engagement and comprehension, promoting patient confidence, utilizing evidence-based content, and delivering material relevant to the medium. The DVD will convey psycho-educational material useful to all parents. Content will be demonstrated through consumer interviews and evidence-based commentary from health professionals. It is estimated that four or five families (parent with cancer, +/− co-parent +/− children) will take part in the DVD. All consumers and health professionals participating in the DVD will be asked to sign a media release and consent form.
- Based on the empirical literature and clinical experience, a QPL to accompany the DVD will be developed by the multidisciplinary research team and consumers on the study steering committee.
- The DVD and QPL will be evaluated for perceived usefulness and acceptability by a sample of consumers and oncology health professionals (approximately *n* = 20). Invited consumers and health professionals will have the option of evaluating the DVD and QPL remotely via online platform or by attending a screening event in person. Collaborating community-based organizations will circulate an invitation to the screening to their members, and oncology health professionals will be invited by the research team through hospital and professional networks. The DVD and QPL will be evaluated using a purpose-designed questionnaire and for those attending the viewing in person, also through facilitated group discussion. The questionnaire will ask about demographic information, overall impressions of the DVD and QPL, their content, and perceived usefulness. Modifications to the DVD and QPL will be incorporated after the evaluation.
- The final component of the intervention, the follow-up telephone call, and provision of additional materials, will then be developed. This telephone call will be conducted by a clinical psychologist (LS). The purpose of this telephone call is to review and consolidate learnings from the DVD and QPL and provide further information (verbal and written) or referrals, if needed. The information to be provided if needed will cover topics that are not necessarily covered in depth in the DVD but which may be relevant. This content will be developed in the form of information sheets which can be mailed or emailed to participants.

Phase 2 of the MRC framework comprises *piloting the feasibility of the intervention*.

### Design

In phase 2, the intervention will be evaluated using a single arm, pre-test post-test design in 20 families where one parent has received treatment for cancer. A sample size of 20 was chosen based on practical considerations including participant flow, budgetary constraints, and the number of participants needed to reasonably evaluate the feasibility and acceptability of the intervention.

### Sample

The sample is divided into two subsamples.

- *Subsample 1* comprises parents with a cancer diagnosis. Participants may be male or female and with any type of cancer.
  Inclusion criteria: parenting at least one child aged 3–12 years old, ability to provide informed consent, ability to complete questionnaires and understand the DVD in English, age 18 years or older, diagnosed with cancer in the past 6 months, and be receiving treatment with curative intent or with a view to longer term survival.
  We intend to recruit 20 parents with cancer. Parents with cancer have the option of participating in the study with or without a co-parent (subsample 2).
- *Subsample 2* comprises co-parents of the person with a cancer diagnosis. A co-parent is another adult involved in the parenting of the children and need not necessarily be a biological parent.
  Inclusion criteria: co-parenting at least one child aged 3–12 years old, the ability to provide informed consent, ability to complete questionnaires and understand the DVD in English, and age 18 years or older.
  Co-parents will have the option of participating in the study without the parent with cancer.

### Procedures

#### Ethical approval

The study has received institutional multisite ethics approval from the Melbourne Health Research Ethics Committee (reference number HREC/16/MH/183).

### Recruitment

*Subsample 1* will be recruited from cancer services across three major tertiary facilities in metropolitan Melbourne, Australia. Subsample 1 will be identified as potentially eligible for recruitment by their treating clinicians (e.g., physician, nurse, social worker) and will be asked to provide consent to be contacted by the study research assistant. Clinicians will complete a referral form noting the patients’ contact and clinical details to be actioned by the research assistant.

To assess the feasibility of this intervention and to describe the participating sample, clinical, and demographic details of all potential participants approached to take part will be collected so that the characteristics of those who decline and any identified reason for declining participation can be recorded. In addition, the clinical and demographic details of those participants who cannot consent due to mental impairment or who do not speak English but who would otherwise be eligible for participation will also be collected. Once in contact with parents who opt in, the study research assistant will explain the nature, scope, and purpose of the study and describe the commitments involved in participating according to a script. If the potential participant provides verbal consent to participate in the study, the research assistant will mail study information and the consent form for the participant to sign and send back in a reply paid envelope.

*Subsample 2* will be identified via subsample 1. The research assistant will, if appropriate, invite the parent with cancer to share details of the study with the co-parent. Once identified by the parent with cancer, the co-parent may participate in the study even if the parent does not wish to participate. The research assistant will explain the nature and purpose of the study to the co-parent separately, as needed. As with subsample 1, if the co-parent provides verbal consent to be in the study, the research assistant will mail the consent form for the participant to sign and send back in a reply paid envelope.

In order for the co-parent to participate in the intervention in the absence of the parent with cancer, the latter is required to provide consent for use of clinical and demographic data.

### Intervention content and delivery

As noted above, the intervention will be delivered via three components developed during phase 1 of the study and will comprise:

- A DVD to convey psycho-educational material relevant to all parents with content demonstrated through consumer interviews and evidence-based commentary from health professionals; accompanied by
- The QPL, which will tailor the intervention to individual needs by bridging the gap between the more broadly applicable information conveyed in the DVD and information of particular relevance to the parent/family [31]; followed by
- A telephone call from a clinical psychologist (LS) 2 weeks later to summarize, review and consolidate progress, and provide additional resources and referrals, if appropriate.

### Data collection

Data will be collected from all participants using validated self-report questionnaires that are administered before the intervention and again 1 month later. Measures are described below.

The pre-intervention questionnaire can be completed electronically or by paper. If completed electronically, a web-link to the survey will be emailed to the participant. If the participant prefers to complete a paper version, the questionnaire can be mailed to them with a reply paid envelope included for returning the questionnaire. The questionnaire will be posted, or emailed with a web-link, to participants 2 weeks prior to receiving the DVD and QPL. DVDs and QPLs will be mailed to participants or will be accessible via online platform (in password protected form). Post-intervention questionnaires will be mailed to participants (with reply paid envelope included) or completed electronically (a web-link to the questionnaire emailed) 4 weeks after the telephone follow-up call. If questionnaires have not been completed or received 2 weeks after being sent, the study research assistant will telephone participants or send a reminder letter/email. Questionnaires will take approximately 30 min to complete.

Participants who score above recognized thresholds on the self-report measures of depression and anxiety will be notified that we will contact them to discuss referral for psychological support.

### Measures

The questionnaires administered pre- and post-intervention will assess parenting stress, parental psychological morbidity, quality of life, family functioning, parenting self-efficacy, and parental perceptions of child adjustment.

*Parental psychological morbidity* will be measured with the Depression Anxiety Stress Scale Short Form (DASS-21 [34]). The DASS-21 is a set of three self-report scales designed to measure the negative emotional states of depression, anxiety, and stress. Participants are asked to use 4-point severity/frequency scales to rate the extent to which they have experienced each state *over the past week*. Higher scores on this 21-item instrument indicate greater symptom burden. Internal consistency and concurrent validity of the DASS-21 have been confirmed [34, 35].

*Parenting stress* will be measured with the Revised Parenting Stress Index Short Form (PSI-R SF) [36]; a 36-item scale comprising 3 domains: parental distress, parent-child dysfunctional interaction, and difficult child, all of which combine to form a Total Stress Scale. Reliability and validity of the scale are well established. Parents respond to each statement using a 5-point scale to indicate the degree to which that item has been disturbing to them in the past week. Parents who obtain a Total Stress score above a raw score of 90 are considered to experiencing clinically significant parenting stress. Internal consistency reliability for the composite Total Stress is reported by the author to be .91. Stability of the instrument was assessed by test-retest after a 6-month interval and yielded an alpha of .84 for the Total Stress [37].

*Parenting self-efficacy and satisfaction* will be assessed using both a general measure, the Parental Sense of Competence Scale (PSOCS) [38]; and a scale that has been developed specifically for use in parents with cancer, the *Cancer-Related Parenting Self-Efficacy s*cale (CaPSE) [39]. The PSOCS comprises 17 items and is scored on a 6-point Likert scale from “strongly agree” to “strongly disagree.” There is good evidence for its factor structure and validity. The CaPSE comprises 24 items that are specifically relevant to parents with cancer. Items are rated on a 6-point Likert scale ranging from strongly disagree to strongly agree for how confident parents feel about their ability to perform certain tasks. Adequate reliability and validity have been reported [39].

*Family functioning* will be measured with the General Functioning (12 items) and Communication (6 items) subscales of the Family Assessment Device (FAD) [40], a highly validated and extensively used instrument based on the McMaster Model of Family Functioning [40, 41]. The FAD assesses structural and organizational properties of families and the patterns of transactions among family members. Responses are scored on a 4-point Likert scale from strongly agree to strongly disagree.

*Concerns about parenting in the context of cancer* will be measured with the cancer-specific Parenting Concerns Questionnaire [4], a 15-item instrument. Items are scored on a 5-point Likert scale of “not at all concerned” to “extremely concerned.” The scale has three subscales: practical impact of the illness on children, emotional impact of the illness on children, and concerns about the co-parent. The scale has shown adequate reliability and validity [4].

*Parental perceptions of the behavioral functioning of children* will be measured with the Strengths and Difficulties Questionnaire (SDQ [42]). The SDQ asks about 25 attributes that are divided between 5 scales: emotional symptoms, conduct problems, hyperactivity/inattention, peer relationship problems, and prosocial behavior. The same 25 items are included in questionnaires for completion by the parents of 4–16 year olds [42]. For parents of 3- and 4-year-old children, the questionnaire is slightly modified: 22 items are identical, the item on reflectiveness is softened, and items on antisocial behavior are replaced by items on oppositionality. The SDQ includes a follow-up questionnaire and additional impact questions which are ideal for use following an intervention such as the one proposed in this study. In cases where participants have multiple children, they will be asked to complete this section with reference to the child about whom they are the most concerned.

*Quality of life* will be measured with the Functional Assessment of Cancer Therapy-General (FACT-G [43]) in subsample 1 and the Functional Assessment of Cancer Therapy-General Population (FACT-GP [44]) in subsample 2. The FACT-G was developed specifically for cancer patients. Scores on both the FACT-G and FACT-GP are a composite of subscales relating to physical, social, emotional, and functional well-being. Higher scores indicate better well-being. Reliability and validity of the FACT-G and FACT-GP have been well documented [43, 44].

*Relevant clinical and sociodemographic data* will be collected from participants including date of birth; gender; number of children; ages of children; relationship status; living arrangements, educational status, and annual income; residential postcode and employment status; mental health treatment history; presence of major medical comorbidities; time since cancer treatment commenced; nature of cancer diagnosis (tumor stream and stage); and nature of cancer treatment. In the event of missing or inconsistent data, participants’ medical records will be checked. Participants will provide consent for access to their medical records.

### Feasibility of the intervention

Feasibility and acceptability of the intervention will be determined by participant evaluation of the acceptability, perceived usefulness, and benefits of the intervention. We will assess participant perceptions of the content, structure, timing, and delivery of the intervention as part of the data collected post-intervention. All participants will complete an evaluation of the intervention as part of the questionnaire administered post-intervention as well as complete a semi-structured telephone interview. With participant consent, this telephone interview will be audiorecorded for later analysis. Interviews will be downloaded from a link emailed to the researchers and saved under the participant’s unique study-assigned identifier. No identifiable information will be voiced on the recording. Data on rates of referral, consent, participation, and attrition for parents and co-parents, as well as reasons for these rates, will be collected wherever possible.

### Analysis

Quantitative data will be analyzed using SPSS version 23.0. Descriptive statistics will be conducted on the measures of parenting stress, parenting concerns, quality of life, self-efficacy and psychological morbidity; family functioning and perceptions of child behavioral functioning. Qualitative data from the phase two telephone interviews conducted to assess participants’ views of the acceptability and benefits of the intervention will be analyzed by repeated listening of recordings and organization of notes into related areas of concern or positive experience [45].

## Discussion

The impact of parental cancer on children and families has gained prominence in recent years and there is increasing recognition of the need for psychosocial interventions to provide psycho-education, emotional support, and reassurance about parenting competence. Yet, intervention research lags far behind descriptive research in this field. A small number of interventions have been published, but with their extensive infrastructure demands, none are suitable for routine use in most healthcare settings. For most developed countries, the healthcare environment is characterized by rising costs, workforce shortages, and the need to provide effective, sustainable services with minimal burden on infrastructure. There is an increasing desire in healthcare organizations for care to be delivered through low-intensity, targeted interventions that can be effectively delivered by combining technology and human contact rather than relying on either in isolation [46]. There is also growing recognition of the importance of patient autonomy and self-management [47].

The current study is appropriate to this context in several ways. First, developing an audiovisual resource as the predominant vehicle to impart general psycho-education constitutes recognition that patients need access to disease-management resources from outside of the hospital setting [46]. Audiovisual media are relatively inexpensive and highly accessible and can convey intense, focused information efficiently through repeated viewing. Such material has been used successfully in cancer settings to provide information about a range of treatment- and survivorship-related issues [48]. Second, QPLs are inexpensive to implement into routine care and effectively encourage patient-clinician communication [49], which, in turn improves psychosocial and physical health outcomes [50, 51]. QPLs also enhance patient autonomy and support a model of tailored, patient-centered care by directing consultation towards issues that specifically concern the patient [31]. The use of a QPL in the current intervention will serve to connect the more general psycho-education imparted in the DVD with issues of specific relevance to a given parent or family. Third, by providing clinical contact by phone call rather than in the form of face-to-face, hospital-based individual or family consultation, the intervention can be said to be low-intensity and tailored to individual needs. The intervention is further tailored to unique needs by adopting a stepped care approach of providing additional resources and referrals at this final stage of the intervention, if they are needed. Finally, the proposed intervention is suitable to the current healthcare environment in that it will comprise an easily distributed and accessible program that can be implemented across cancer services and offered in acute settings wherever cancer care is delivered.

We acknowledge several limitations of the scope of this work. The intervention is focused on parents receiving curative cancer treatment or treatment with a view to longer term survival and will not be suitable for parents who have a limited life expectancy as their needs and the needs of their children differ from those of the target population. The intervention content is tailored for parents with pre-adolescent children and while general principles communicated will be largely applicable to children of any age, the program is less useful for parents with adolescent children. Finally, the intervention is aimed at parents only and does not involve direct support or counseling of children or direct coaching of the parent-child interaction. Limitations aside, this intervention will meet an expressed need for an evidence-based, accessible psycho-educational intervention to support, empower, and educate parents with cancer who have children aged 3–12 years. In so far as few oncology professionals are specifically trained in managing psychosocial aspects of parental cancer, the intervention will support clinical care more generally. Finally, this intervention will advance the field by providing a template for the development of similar interventions in this area differentiated by the developmental stage of the dependent child (e.g., adolescent children) and the information needs of the targeted parent/patient (e.g., parents with genetic predisposition to cancer).

## Abbreviations

CaPSE: Cancer-Related Parenting Self-Efficacy Scale
DASS-21: Depression Anxiety Stress Scale Short Form
FACT-G: Functional Assessment of Cancer Therapy-General
FACT-GP: Functional Assessment of Cancer Therapy-General Population
FAD: Family Assessment Device
MRC: Medical Research Council
PSI-R SF: Revised Parenting Stress Index Short Form
PSOCS: Parental Sense of Competence Scale
QPL: Question Prompt List
SDQ: Strengths and Difficulties Questionnaire
UK: United Kingdom
